# Editorial Comment: Effect of Behavioral and Pelvic Floor Muscle Therapy Combined With Surgery vs Surgery Alone on Incontinence Symptoms Among Women With Mixed Urinary Incontinence: The ESTEEM Randomized Clinical Trial

**DOI:** 10.1590/S1677-5538.IBJU.2020.04.12

**Published:** 2020-05-20

**Authors:** Cássio L. Z. Riccetto

**Affiliations:** 1 Divisão de Urologia Feminina Faculdade de Ciências Médicas Universidade Estadual de Campinas CampinasSP Brasil Divisão de Urologia Feminina - Faculdade de Ciências Médicas da Universidade Estadual de Campinas – UNICAMP, Campinas, SP, Brasil

Sung VW ^1^, Borello-France D ^2^, Newman DK ^3^, Richter HE ^4^, Lukacz ES ^5^, Moalli P ^6^, et al.

^1^ The Division of Urogynecology and Reconstructive Pelvic Surgery, Department of Obstetrics and Gynecology, Alpert Medical School of Brown University, Providence, Rhode Island; ^2^ Department of Physical Therapy, Rangos School of Health Sciences, Duquesne University, Pittsburgh, Pennsylvania; ^3^ The Division of Urology, Department of Surgery, Perelman School of Medicine, University of Pennsylvania, Philadelphia; ^4^ Division of Urogynecology and Pelvic Reconstructive Surgery, Department of Obstetrics and Gynecology, University of Alabama at Birmingham; ^5^ The Division of Female Pelvic Medicine & Reconstructive Surgery, Department of Obstetrics, Gynecology & Reproductive Sciences, University of California San Diego, La Jolla; ^6^ Women’s Center for Bladder and Pelvic Health, Division of Urogynecology and Reconstructive Pelvic Surgery, Department of Obstetrics, Gynecology and Reproductive Sciences, University of Pittsburgh Medical Center, Pittsburgh, Pennsylvania

JAMA. 2019 Sep 17;322(11):1066-1076

**DOI: 10.1001/jama.2019.12467 | ACCESS: 10.1001/jama.2019.12467**

## COMMENT

The use of physical therapy has been strongly recommended in the most popular guidelines regarding stress urinary incontinence ( 1 ) and overactive bladder ( 2 ). In this interesting article published in JAMA, the authors presented a prospective randomized study that sought to evaluate whether performing a mid-urethral sling combined with behavioral therapy and 6 pelvic floor muscle training (PFMT) sessions (n: 209) would yield better outcomes compared to exclusive mid-urethral sling implant (n: 207) in patients with mixed urinary incontinence. Patients were evaluated by the long-form Urogenital Distress Inventory (UDI), and the primary outcome was defined as significant improvement over the baseline condition after 12 months post treatment. In the group that performed the behavioral plus TMAP plus sling, the UDI score decreased from 178.0 points to 30.7 points (adjusted mean change −128.1 points - 95% CI, −146.5 to −109.8). In the group that was treated by sling alone, the score decreased from 176.8 to 34.5 points (adjusted mean change −114.7 points - 95% CI, −133.3 to −96.2). The model-estimated between-group difference (−13.4 points; 95% CI, −25.9 to −1.0; P = .04) did not meet the minimal clinically important difference threshold. The authors concluded that in women with mixed urinary incontinence, the addition of behavioral and TMAP measures to the mid-urethral sling did not determine clinically relevant changes.

Mixed symptoms represent the majority and most challenging subpopulation among those with incontinence ( 3 ). Moreover, even the sling implant can potentially lead to the worsening of storage symptoms in patients with pre-operative mixed urinary incontinence. It is highly unlikely that a unified algorithm could be applicable to all patients, due to the multifactorial origin of the symptoms. Although we agree to the multimodal approach for mixed incontinence, studies that clearly address the cost-effectiveness of combined treatments are still lacking.
